# Liraglutide suppresses obesity and induces brown fat-like phenotype via Soluble Guanylyl Cyclase mediated pathway *in vivo* and *in vitro*

**DOI:** 10.18632/oncotarget.13189

**Published:** 2016-11-07

**Authors:** Endong Zhu, Yang Yang, Juanjuan Zhang, Yongmei Li, Chunjun Li, Liming Chen, Bei Sun

**Affiliations:** ^1^ Key Laboratory of Hormones and Development (Ministry of Health), Tianjin Key Laboratory of Metabolic Diseases, Tianjin Metabolic Diseases Hospital and Tianjin Institute of Endocrinology, Tianjin Medical University, 300070 Tianjin, China; ^2^ Department of Human Anatomy and Histology, Tianjin Medical University, 300070 Tianjin, China

**Keywords:** liraglutide, white fat browning, obesity, soluble guanylyl cyclase, uncoupling protein 1

## Abstract

Strategies for driving white adipose tissue (WAT) to acquire brown-like characteristics are a promising approach to reduce obesity. Liraglutide has been reported to active brown adipose tissue (BAT) thermogenesis and WAT browning by rapid intracerebroventricular injection in mice. In this study, we investigated the effects and possible mechanisms of liraglutide on WAT browning by chronic treatment. Here, we show that liraglutide significantly decreases body weight of mice and reduces the size of white adipocytes. By quantity polymerase chain reaction, immunoblotting analysis, cell immunofluorescence or immunocytochemical staining, we found liraglutide induced WAT browning because it up-regulated lipolytic activity, BAT, as well as mitochondrial marker genes in inguinal and peripheral renal WAT. We also confirmed liraglutide induced browning of 3T3-L1 because it enhanced expression of BAT and mitochondrial specific genes. In further, we observed that, soluble guanylyl cyclase (sGC) and protein kinase G I (PKGI) were up-regulated by liraglutide *in vivo* and *in vitro*; stimulation of sGC elevated expression of BAT markers and PKGI, which suggested that liraglutide induced WAT browning via sGC-dependent pathway. Taken together, this study expands our knowledge on the mechanism of liraglutide inducing WAT browning, and provides a theoretical support for clinical usage of liraglutide on obesity treatment.

## INTRODUCTION

Obesity has become a major worldwide health challenge, cause of the admitted health risks and sustained increase in prevalence [1]. For decades, as the most common metabolic disorder, it affects hormones and cytokines secretion as well as results in a chronic inflammatory condition, leading to numerous diseases, including cardiovascular disease, type 2 diabetes mellitus (T2DM), hypertension, certain cancers and so on [2–4]. Obesity is defined as the accumulation of excess adiposity resulting from energy intake exceeding energy consumption [5, 6]. The first considered treatment for fighting against diet-induced obesity is reducing food intake or enhancing exercise. However, these methods can't control the increasing trend of obesity sufficiently, so novel therapeutic strategies should be searched to relieve the healthy impact of obesity and its comorbidities.

It is well known recently that there are mainly two adipose tissues in mammals, white adipose tissue (WAT) and brown adipose tissue (BAT). WAT is a critical depot for energy storage, and the predominant constituent is white adipocyte containing a single big fat droplet and less mitochondria; in contrast, the major composition of BAT is abundant brown adipocyte, which is characterized by greater numbers of small fat droplets and mitochondria [7]. Moreover, BAT is specialized for providing thermogenesis, which is mediated by uncoupling cellular respiration using uncoupling protein 1 (UCP1) in the inner mitochondrial membrane [7, 8]. UCP1 plays a crucial role in allowing electrons to be released rather than stored, resulting in heat release. UCP1 deficient mice displayed an impaired ability to produce heat by non-shivering thermogenesis, exhibited cold intolerance, and gained more body weight than wild-type controls [9, 10], so BAT activation had emerged as an attractive therapeutic target for the treatment of obesity [11]. After many years of research, intriguingly, a third type of adipocytes has been discovered in rodent and human, which is called brown-fat like adipocytes (also termed inducible brown, brite or beige adipocytes) that disperses in white fat depots, similar to brown adipocytes, which express UCP1 and other thermogenic genes to dissipate energy [8, 12]. In this regard, more and more scientific literatures have focused on WAT browning because of its beneficial effects in the thermogenic property, and it may be another highly suitable therapy to combat obesity [7, 13, 14]. Several mediators have been reported to involve in the process of WAT browning, including adrenergic stimulation, intermittent cold exposure, exercise, and hormonal stimuli [7]. Based on these mediators, a number of transcriptional regulators and related regulatory signaling pathways were identified [15], three of which were considered as the key nodes in the regulation of inducible brown fat [15], namely PR domain-containing 16 (PRDM16) [16], peroxisome proliferator-activated receptor γ (PPARγ) [17] and peroxisome proliferator-activated receptor gamma co-activator 1α (PGC1α) [18]. For transmitting hormonal signals from receptors on the cell membrane into the endochylema in adipocytes, second messengers cyclic adenosine monophosphate (cAMP) and cyclic guanosine monophosphate (cGMP) are important; cAMP-initiated lipolysis results in the release of free fatty acids and activation of UCP1 via cAMP/PKA pathway; cGMP, synthesized by nitric oxide (NO)-sensitive soluble guanylyl cyclase (sGC), induces mitochondrial biogenesis and increases UCP-1 abundance depending on protein kinase G I (PKGI) [19]. Recently, increasing numbers of agents treating obesity and diabetes were found to have beneficial effect on BAT activation and/or WAT browning, but the detailed mechanisms are not well understood.

Glucagon-like peptide-1 (GLP-1) is an incretin hormone that is released from enteroendocrine L-cells located in the ileum and colon in response to caloric intake [20]. GLP-1 activates the GLP-1 receptor (GLP-1R), leading to stimulating insulin secretion, biosynthesis and sensitivity, improving the damage of β-cell, reducing glucagon secretion, slowing gastric emptying as well as suppressing appetite [21]. Based on above functions of GLP-1, liraglutide, as a GLP-1 long-lasting analogue, has been used for the treatment of T2DM, which, besides its well-known anti-diabetic properties, has been also recommended for the treatment of obesity [22]. Native GLP-1 is a 30-amino acid peptide. For improving its stability, liraglutide has structural modifications including substitution of Lys34 to Arg and addition of a C16 fatty acid at position 26 using a γ-glutamic acid spacer, which increases the half-life to 20h helping to amend pharmacokinetics for once-daily administration without compromising biological activity [23]. Liraglutide may help to combat obesity by its contribution of BAT activity. GLP-1 and GLP-1R are abundant in areas of the central nervous system (CNS), and the sympathetic nervous system (SNS) is essential for control of BAT metabolism by CNS [24]. Recent evidence has shown that CNS-GLP-1R signaling directly stimulates the interscapular BAT thermogenesis in mice [24]. Another group reported that central GLP-1 receptor signaling accelerates plasma clearance of triacylglycerol (TAG) and glucose by activating BAT in mice [25]. To further explore the molecular mechanisms of the core thermoregulatory network involved in the actions of GLP-1, Beiroa et al. found that after intracerebroventricular (ICV) injection of liraglutide, the brain GLP-1 system increased the thermogenic activation of BAT through hypothalamic AMP-activated protein kinase (AMPK) located in the hypothalamic ventromedial nucleus [26]. Recently, studies regarding GLP-1 in adipose tissue mainly focus on the BAT activation, and the possible mechanisms of GLP-1 on the WAT browning remains unclear.

In this report, we show that body weight loss of mice was induced by chronic peripheral treatment with the GLP-1 analogue, liraglutide. We found that liraglutide up-regulated BAT and mitochondrial biogenic marker genes in WAT and in adipocyte-inducing 3T3-L1 cells. sGCβ-mediated pathway was confirmed involving in the above process. Our findings indicate that liraglutide induces WAT browning through sGCβ dependent pathway.

## RESULTS

### Liraglutide reduces obesity in HFD-fed KKAy mice

**Figure 1:**
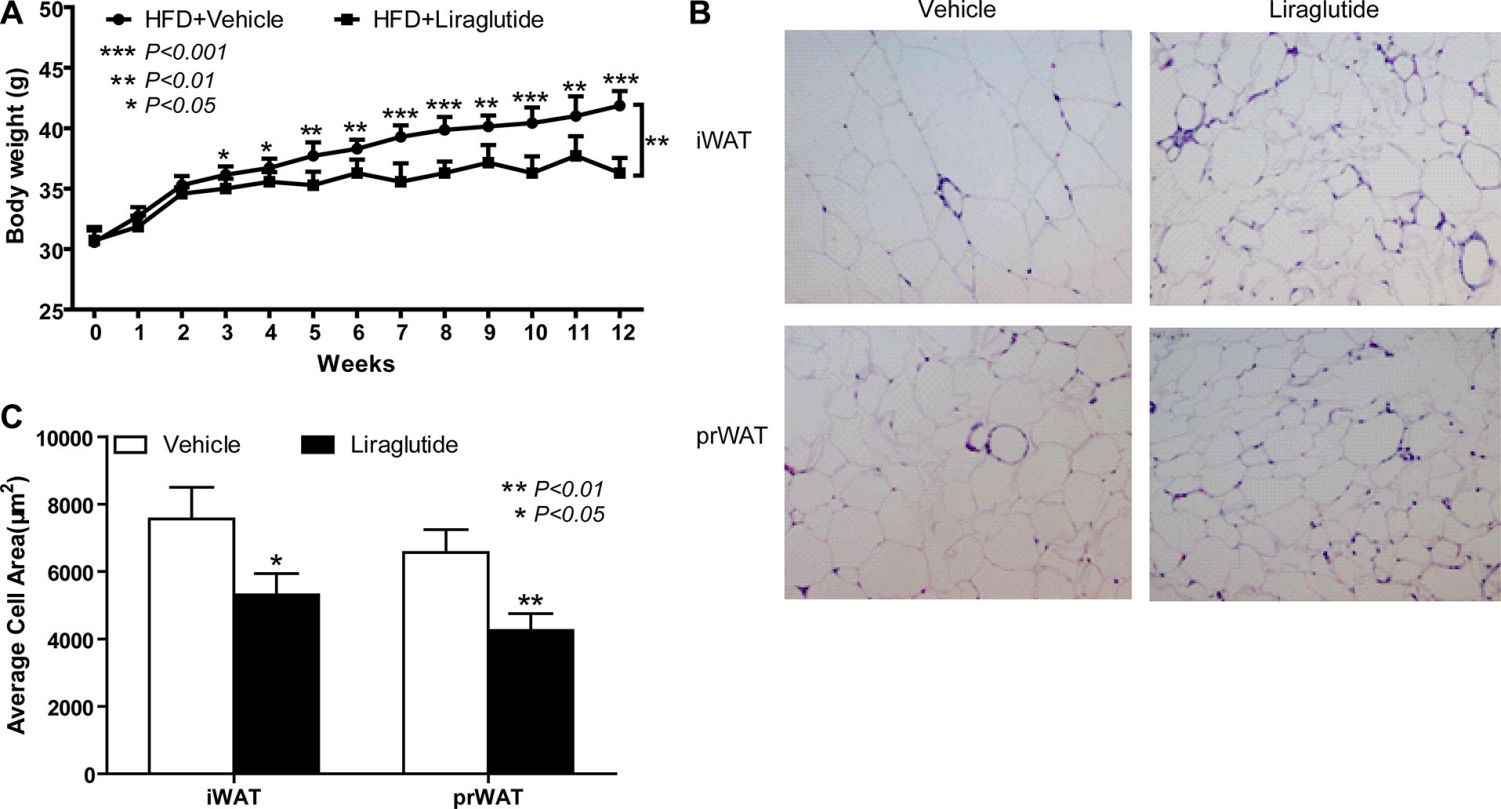
Liraglutide reduces obesity in HFD-fed KKA mice (**A**) 8-week-old male HFD-fed KKAy mice were randomized to be injected once daily with either vehicle (PBS) or liraglutide (final dose 400 μg/Kg body weight). The body weight of mice was recorded every day. Data are representative of three independent experiments. HFD+Vehicle, *n* = 7, HFD+Liraglutide, *n* = 7; the values are means ± S.D. *P < 0.05, **P < 0.01, ***P < 0.001, compared with the HFD+Vehicle group (Student's *t* test in everyday body weight statistics and ANOVA followed by Bonferroni multiple comparison pos*t*-tests to analysis of variance in two groups). (**B**) Representative H&E staining of iWAT and prWAT sections from HFD+Vehicle mice and HFD+Liraglutide mice respectively treated with PBS or liraglutide after 12 weeks. Image magnification: 100×. Data are shown from one of three independent experiments. (**C**) Adipocyte size in B was determined by computer image analysis as described in “Materials and Methods”. Data are shown from one of three independent experiments, the values are means ± S.D. *P < 0.05, **P < 0.01, compared with the HFD+Vehicle group.

Numerous studies reported that the body weight could be controlled by the use of liraglutide in human and mice [27–31]. To certify the function of liraglutide in obesity, the high fat diet (HFD)-fed KK/Upj-Ay/J (KKAy) mouse was used. Male mice were randomized to be subcutaneously (s.c.) injected once daily with either vehicle (PBS) or liraglutide (final dose 400 μg/Kg body weight). Similar to what has been reported [29–31], the treatment of liraglutide was able to slow down the body weight gain (Figure 1A). From the beginning of the third week, there was significant difference in the body weight between vehicle- and liraglutide-treated mice; at the end, 12-week liraglutide treatment decreased body weight gain by approximately 13.3% vs. vehicle treated group (*P* < 0.001, Figure 1A). Histomorphological analysis revealed smaller lipid droplets in inguinal WAT (iWAT) and peripheral renal WAT (prWAT) from mice 12 weeks after once daily injection of liraglutide (Figure 1B). The adipocyte surface area of liraglutide-treated group was significantly smaller than vehicle-treated group (iWAT, 7562.2 ± 937.9 μm^2^ vs. 5313.4 ± 623.7 μm^2^, *P* < 0.05; prWAT, 6566.1 ± 676.2 μm^2^ vs. 4254.5 ± 499.5 μm^2^, *P* < 0.01) (Figure 1C). Because of the morphological differences of lipid droplets in iWAT and prWAT between the liraglutide-treated mice and the vehicle-treated mice, we thought liraglutide might influence the white fat browning.

### Liraglutide triggers the white fat browning in mice

**Figure 2:**
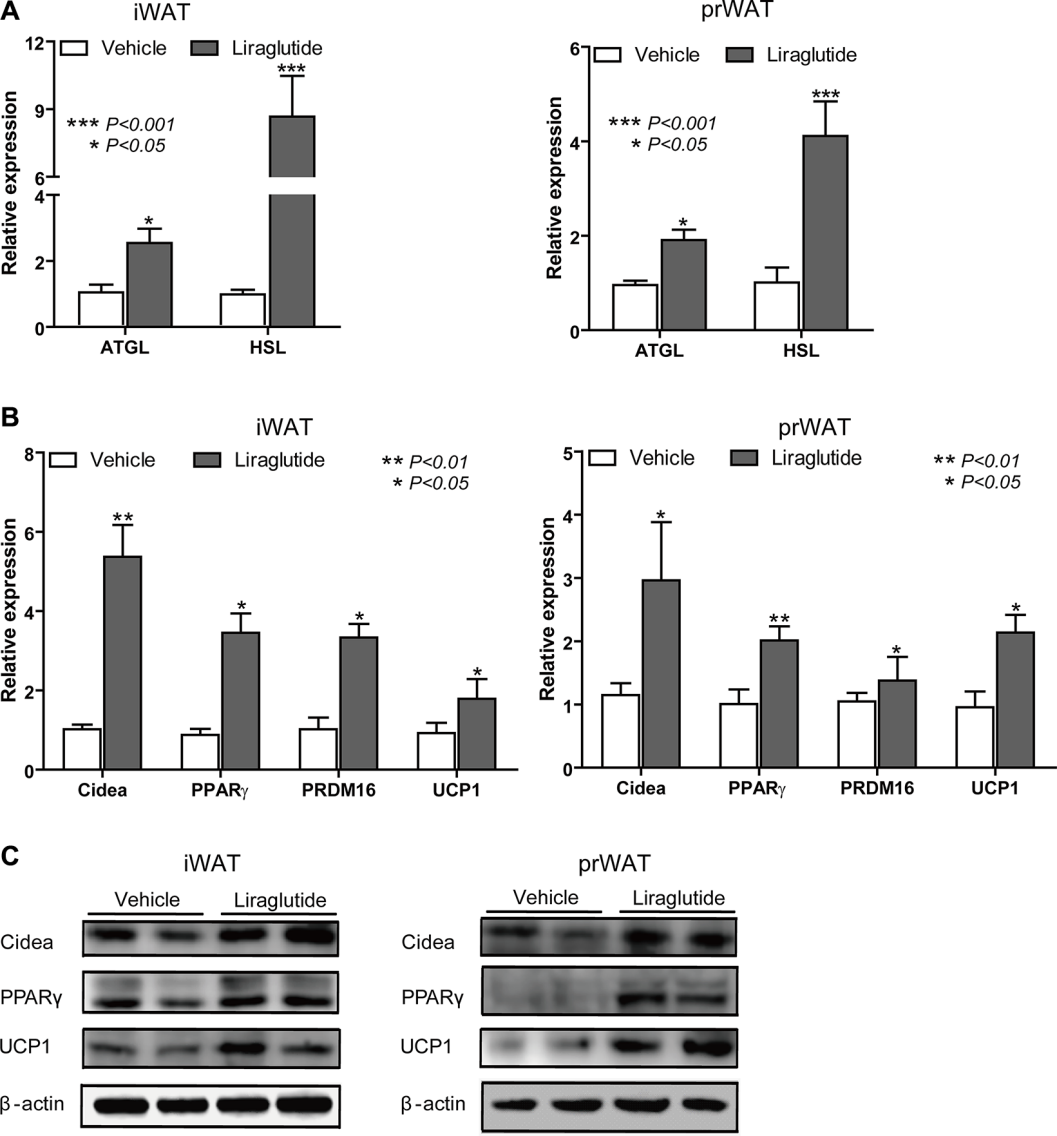
Liraglutide triggers the white fat browning in mice iWAT and prWAT were collected from Vehicle- and Liraglutide-treated HFD-fed mice respectively after 12 weeks. (**A**) Relative expression of lipolytic activity marker genes in iWAT and prWAT was measured by qRT-PCR. (**B**) mRNA expression levels of brown fat marker genes assessed by qRT-PCR on tissue lysates. (**C**) Western blot analysis for Cidea, PPARγ and UCP-1is shown. Above qRT-PCR Results are mean ± S.D., *P < 0.05, **P < 0.01, ***P < 0.001, presented relative to β-actin expression. All data represent three separate experiments.

It has been reported that liraglutide contributes to increase BAT thermogenesis and stimulate BAT activity in mice [24, 26, 31]. In this study, we want to test the WAT browning effect of liraglutide, so the iWAT and prWAT were collected from two groups mice treated as Figure 1A delineated. The relative expression of lipolytic activity marker genes [32] adipose triglyceride lipase (ATGL) and hormone-sensitive lipase (HSL) was examined by qRT-PCR. As shown in Figure 2A, they were significantly up-regulated in iWAT (2.39− and 7.86-fold respectively) and prWAT (1.91− and 3.68-fold respectively) from liraglutide-treated groups. Liraglutide treatment also resulted in great up-regulation of brown fat marker genes [7] cell death-inducing DFFA-like effector A (Cidea), PPARγ, PRDM16 and UCP-1 (5.31−, 3.92−, 3.26− and 1.93-fold respectively to iWAT; 2.57−, 2.01−, 1.31− and 2.23-fold respectively to prWAT) on the mRNA levels expression against vehicle control (Figure 2B). Increased expression of Cidea, PPARγ and UCP1 was further confirmed by Western blot analysis (Figure 2C). These data suggested that liraglutide might induce browning in WAT.

### Liraglutide stimulates mitochondrial biogenesis in WAT

**Figure 3:**
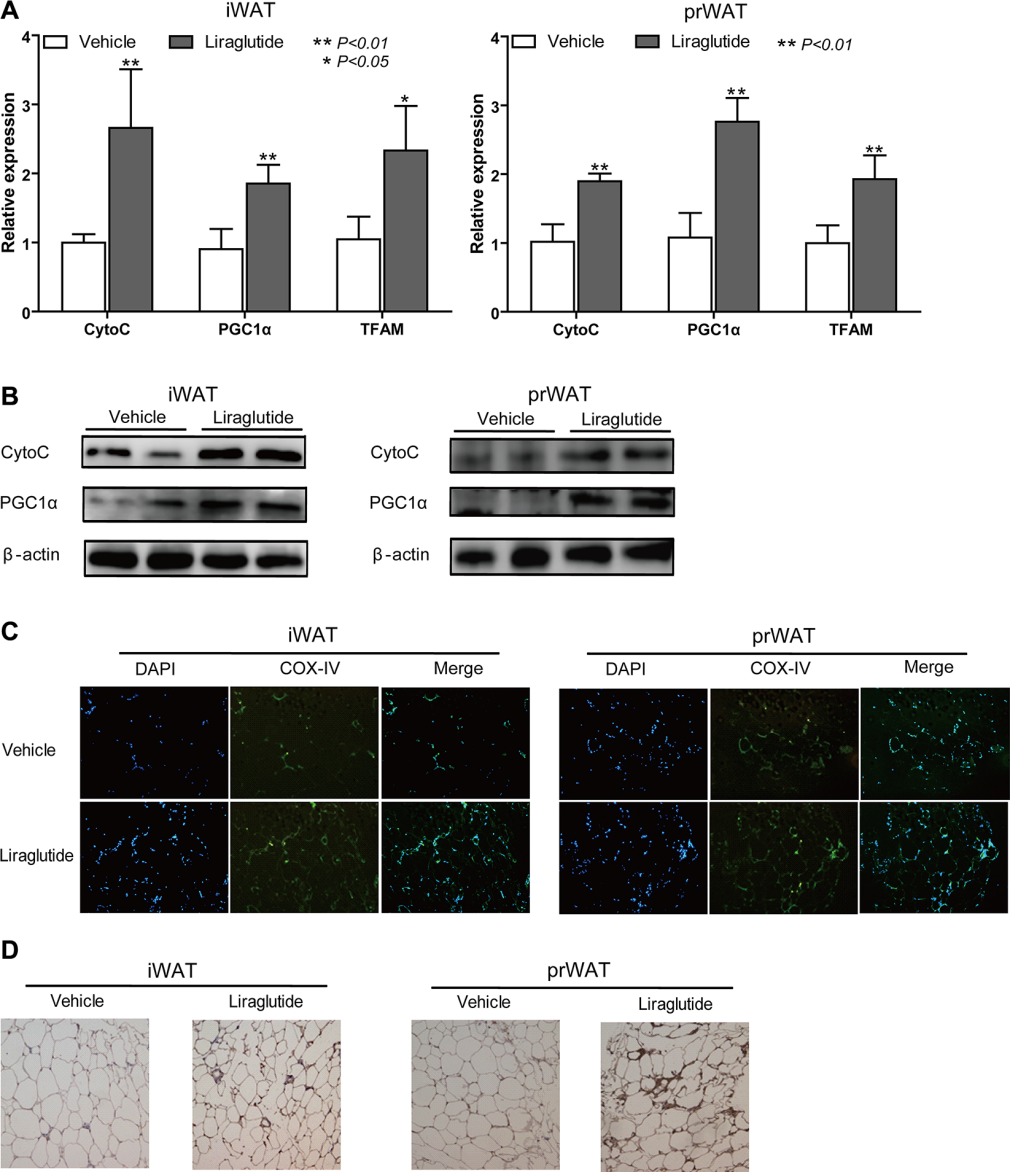
Liraglutide stimulates mitochondrial biogenesis in WAT iWAT and prWAT were collected from Vehicle- and Liraglutide-treated HFD-fed mice respectively after 12 weeks. (**A**) Relative mRNA expression levels of mitochondrial associated genes were analyzed by qRT-PCR on tissue lysates. Data represent means ± S.D., *P < 0.05, **P < 0.01, versus control, presented relative to β-actin expression. (**B**) Protein expression of CytoC, PGC1α and TFAM in WAT were analyzed by immunoblotting. (**C**) Immunostaining for mitochondrial marker gene COX-IV abundance in sections of iWAT and prWAT, COX-IV was labeled as green and nuclei of cells were stained as blue. Representative images were shown. (**D**) Representative images of immunohistochemistry for COX-IV (dark brown stain) distribution were shown. Image magnification in (C and D) 100×. All date above represents three separate experiments.

Considering that more mitochondrial biogenesis is a marked feature of fat browning, the relative expression of mitochondrial associated genes cytochrome C (CytoC), PGC1α and mitochondrial transcription factor A (TFAM) was further measured. As shown in Figure 3A, they were remarkably up-regulated 2.66-fold (*P* < 0.01), 2.06-fold (*P* < 0.01), 2.33-fold (*P* < 0.05) respectively in iWAT, and 1.89-fold (*P* < 0.01), 2.76-fold (*P* < 0.01), 1.93-fold (*P* < 0.01) respectively in prWAT from liraglutide- vs. vehicle-treated group. Immunoblotting further confirmed elevated protein expression levels of above genes (Figure 3B). Moreover, using immunostaining and immunohistochemistry (IHC), we observed enhanced expression of another mitochondrial marker gene cytochrome c oxidase subunit IV (COX-IV) (Figure 3C and 3D). Based on these results, we were more confident that liraglutide could activate the white fat browning.

### Liraglutide induces a brown-like phenotype *in vitro*

**Figure 4:**
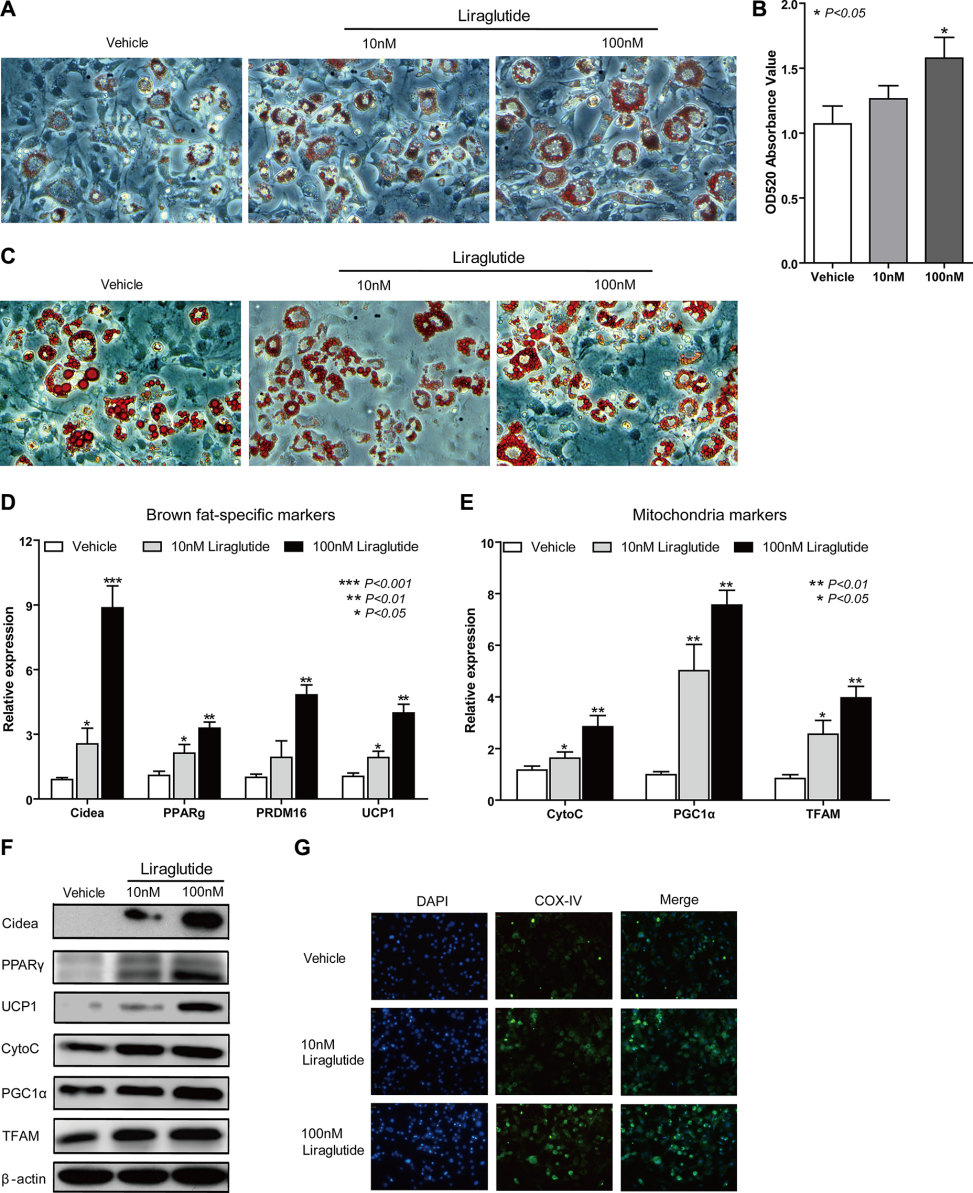
Liraglutide induces a brown-like phenotype *in vitro* (**A**, **B**) 3T3-L1 cells were cultured in adipocyte-inducing medium for 3 days, and then treated with different concentrations of liraglutide (0, 10, 100 μM) in normal culturing medium. Representative images of liraglutide-treated cells at day 3 labeled with Oil Red O (A). Oil Red O extracted with isopropanol was measured at OD520 (B). Fold changes are normalized to the average of 0 μM liraglutide-treated cells. (**C**) Representative images of liraglutide-treated cells as (A) at day 10 labeled with Oil Red O. (**D**, **E**) Liraglutide-treated cells as (A) were collected to extract RNA for testing expression of BAT (D), mitochondrial (E) markers by qRT-PCR. Fold changes are normalized to the average of 0 μM liraglutide-treated cells and are presented relative to β-actin. (**F**) Liraglutide-treated cells as (A) were collected to extract protein for analyzing expression of Cidea, PPARγ, UCP-1, CytoC, PGC1α and TFAM by western blot. (**G**) Immunostaining was used to analyze COX-IV abundance in liraglutide-treated cells as (A) at day 3, COX-IV was labeled as green and nuclei of cells were stained as blue. Representative images were shown. Image magnification in (A and C) 200×, in (G) 100X. All data above are representative at least three independent experiments; the values are means ± S.D. *P < 0.05, **P < 0.01 and ***P < 0.001, versus control.

To further investigate whether liraglutide induces browning *in vitro*, preadipocyte 3T3-L1 was used as a cellular model. For testing the effect of liraglutide on adipogenesis, 3T3-L1 cells were cultured in adipocyte-inducing medium for 3 days, and then treated with different concentrations of liraglutide (10, 100 μM) in normal culturing medium. 100 μM Liraglutide treatment led to a significant increase of lipid droplet numbers after 3 days (25% increase in oil-red O staining, *P* = 0.017) (Figure 4A and 4B). After 10 days' treatment, we observed there were greater numbers of large lipid droplets in vehicle-treated cells, but greater numbers of small lipid droplets in liraglutide-treated cells (Figure 4C). In a dose-dependent manner, liraglutide significantly increased mRNA levels of all brown fat markers examined, including Cidea, PPARγ, PRDM16 and UCP-1 (Figure 4D), as well as mitochondrial markers (CytoC, PGC1α and TFAM) (Figure 4E). Furthermore, enhanced protein expression was certified including Cidea, PPARγ, UCP-1, CytoC, PGC1α and TFAM (Figure 4F). Next, mitochondrial marker gene COX-IV was found to be up-regulated by immunostaining (Figure 4G). Our results indicated liraglutide could increase the brown-like phenotype along with mitochondrial biogenesis *in vitro*.

### Liraglutide activates sGC-dependent pathway

**Figure 5:**
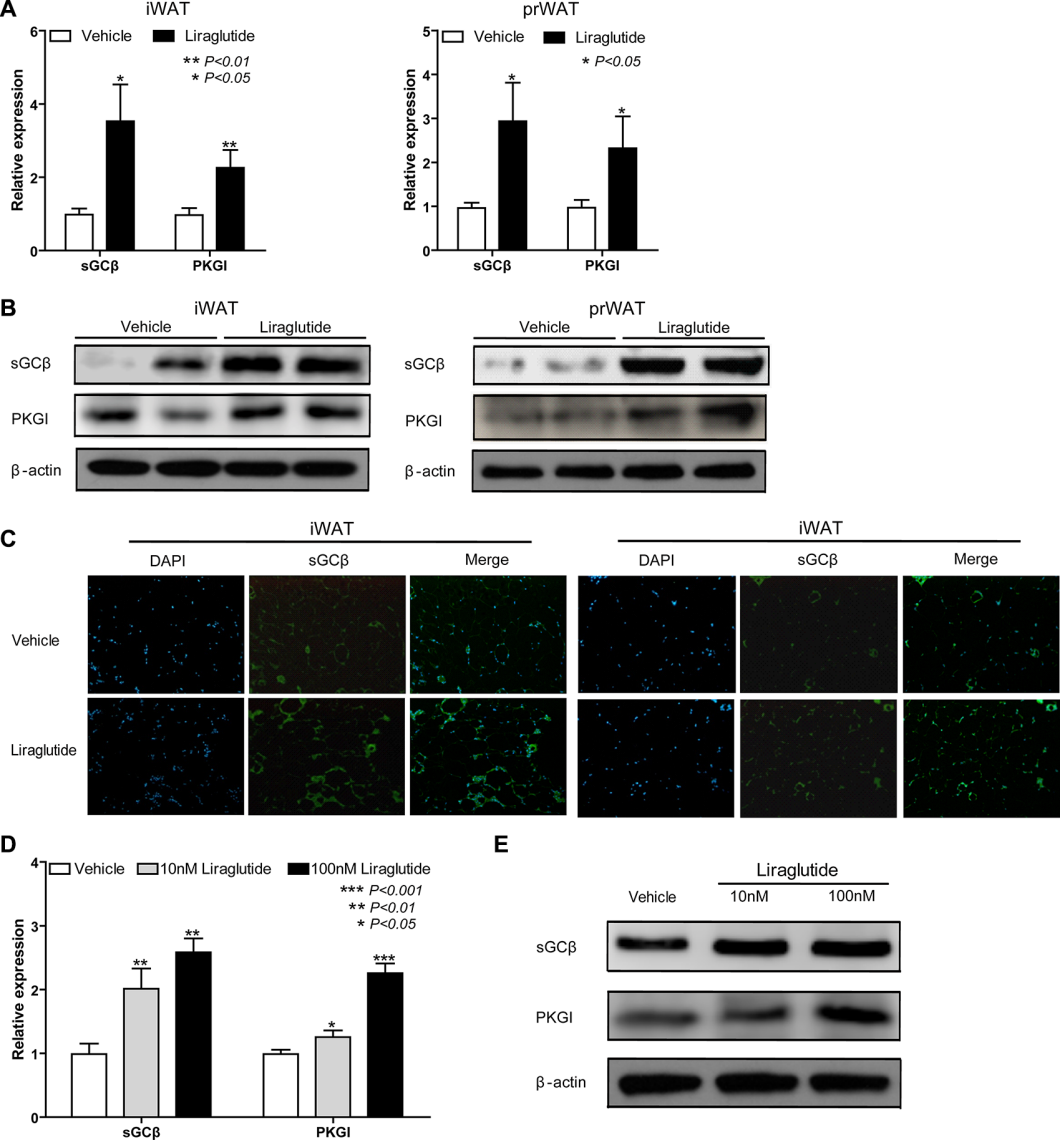
Liraglutide activates sGC-dependent pathway (**A**) iWAT and prWAT were collected from Vehicle- and Liraglutide-treated HFD-fed mice respectively after 12 weeks. As important genes in sGC-dependent pathway involving in mitochondrial biogenesis and brown fat differentiation, the relative expression levels of sGCβ and PKGI in iWAT and prWAT were analyzed by qRT-PCR. (**B**) Protein expression of sGCβ_1_ and PKGI in WAT was analyzed by western blot. (**C**) sGCβ abundance in sections of iWAT and prWAT was tested by immunostaining, sGCβ_1_ was labeled as green and nuclei of cells were stained as blue. Representative images were shown. Image magnification: 100×. (**D**, **E**) 3T3-L1 cells were cultured in adipocyte-inducing medium for 3 days, and then treated with different concentrations of liraglutide (0, 10, 100 μM) in normal culturing medium for 3 days. Cells RNA was extracted to analyze relative mRNA expression levels of sGCβ_1_ and PKGI by qRT-PCR (D). Cells protein was extracted to analyze protein expression of sGCβ and PKGI by immunoblotting. Above qRT-PCR results are mean ± S.D., *P < 0.05, **P < 0.01, ***P < 0.001, Fold changes are normalized to control (vehicle-treated mice or 0 μM liraglutide-treated cells), and presented relative to β-actin expression. All data above are representative of three independent experiments.

We noted that sGC produced the second messenger cGMP, which was a key regulator of mitochondrial biogenesis and brown fat differentiation through directly activated PKGI [33–35]. Therefore, to gain insights into the possible molecular mechanism underlying the browning effect of liraglutide, the expression level of sGCβ and PKGI was tested. As shown in Figure 5A, they were significantly up-regulated 3.60-fold (*P* < 0.05), 2.35-fold (*P* < 0.01) respectively in iWAT, and 3.05-fold (*P* < 0.05), 2.39-fold (*P* < 0.05) respectively in prWAT from liraglutide- vs. vehicle-treated group. The increased protein expression of above genes was confirmed by immunoblotting (Figure 5B). Besides, using the method of immunostaining, the elevated expression level of sGCβ was also to be found. To further explore whether sGCβ_1_ and PKGI were also stimulated by liraglutide *in vitro*, 3T3-L1 cells were treated as Figure 4A described. As shown in Figure 5D and 5E, liraglutide significantly increased mRNA and protein expression levels of sGCβ_1_ and PKGI in a dose-dependent manner. Results above demonstrated that sGC-dependent pathway could be activated by liraglutide *in vivo* and *in vitro*.

### Liraglutide induces brown fat phenotype via sGC-mediated pathway

**Figure 6:**
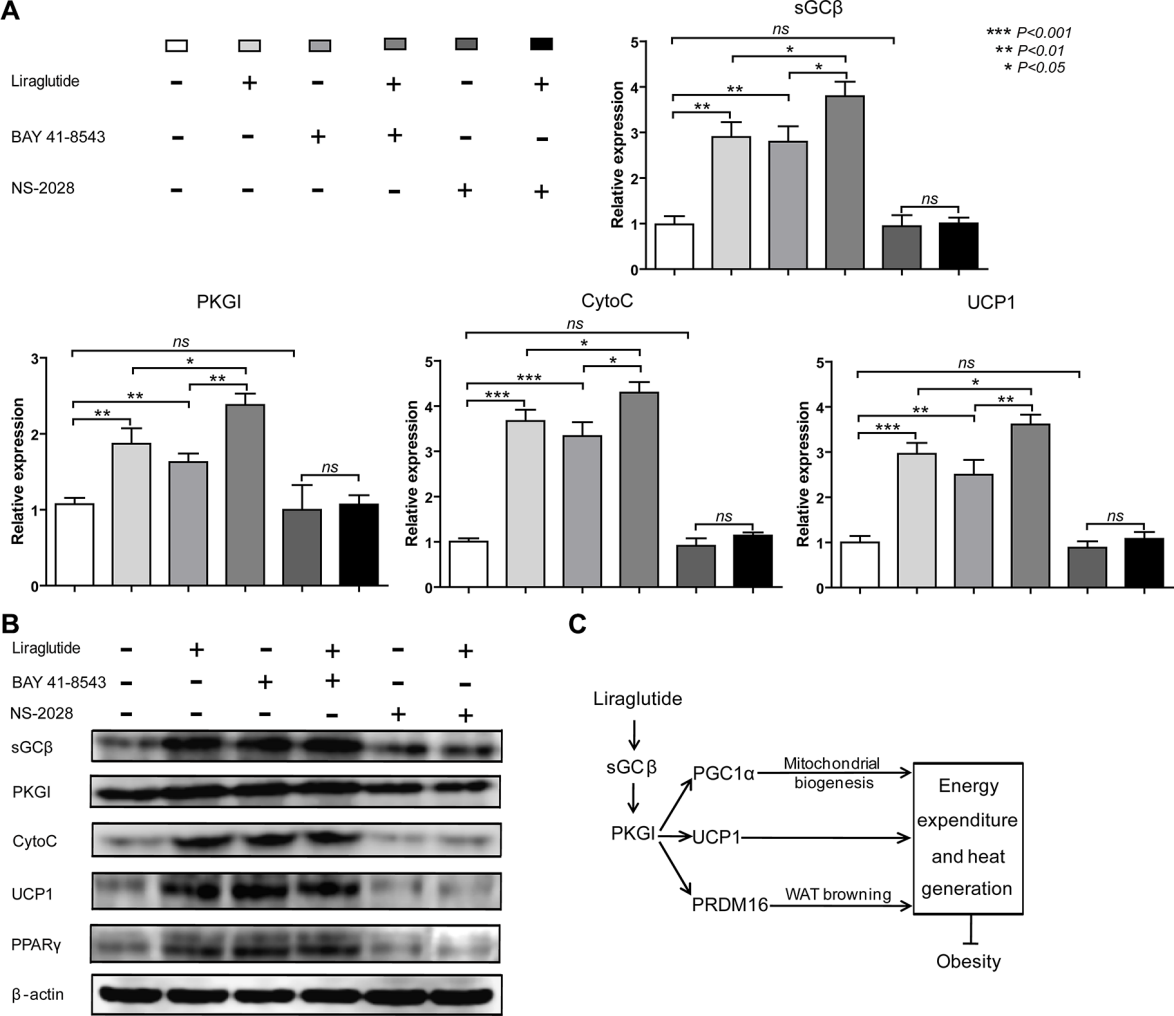
Liraglutide induces brown fat phenotype via sGC-mediated pathway (**A**) 3T3-L1 cells were cultured in adipocyte-inducing medium for 3 days, and then treated with BAY 41-8543 (3 μM) or NS-2028 (100 nM) accompany with adding liraglutide or not. Relative mRNA expression levels of sGC-mediated pathway important genes sGCβ_1_ and PKGI, mitochondrial marker gene CytoC, brown fat marker gene UCP1 was measured by qRT-PCR. The values are mean ± S.D. from three independent experiments, *P < 0.05, **P < 0.01, ***P < 0.001, Fold changes are normalized to no BAY 41-8543, NS-2028 or liraglutide treated cells, and presented relative to β-actin expression. (**B**) Western blot analysis of sGCβ_1_, PKGI, CytoC, UCP1 and PPARγ in cells treated as (A) is shown. Data are representative of three independent experiments. (**C**) Suggested pathway for liraglutide-induced a brown-like phenotype via sGC-mediated pathway, (↓) activation, (┤) inhibition.

The results in Figure 5 drove us to hypothesize that liraglutide induced the brown-like phenotype through the sGC-mediated pathway. To certify our hypothesis, 3T3-L1 cells were cultured in adipocyte-inducing medium for 3 days, and then treated with BAY 41-8543 (3 μM) or NS-2028 (100 nM) accompany with adding liraglutide or not, which is a potent activator and inhibitor of sGC respectively. As shown in Figure 6A, activated expression of sGC by BAY 41-8543 stimulated mRNA expression levels of sGCβ (2.86-fold, *P* < 0.01), PKGI (1.52-fold, *P* < 0.01), CytoC (3.34, *P* < 0.001) and UCP1 (2.51, *P* < 0.01), and adding liraglutide could enhance their expression. However, although there was no statistical difference, suppressed expression of sGC by NS-2028 could slightly reduce mRNA expression levels of sGCβ, PKGI, CytoC and UCP1, and adding liraglutide slightly elevated their expression. The results of protein expression levels were consistent with this (Figure 6B). Our results all supported the idea that activated sGC-mediated pathway potentially involved in the course of liraglutide-induced the white fat browning (Figure 6C).

## DISCUSSION

Numerous evidences have revealed the important role of GLP-1 and its mimetics in control of the body weight gain [21, 36]. In this study, we demonstrate that a GLP-1 analogue, liraglutide, significantly reduces obesity of HFD-fed mice (Figure 1). The previously known mechanisms of liraglutide effect on weight loss are suppression of appetite and inhibition of food intake through hypothalamic or parasympathetic pathways [37]. Recently, BAT activation and WAT browning have emerged as attractive therapeutic targets for the treatment of obesity due to their characteristics of energy expenditure. Accordingly, liraglutide may help to combat obesity through above two pathways. Although several groups have reported that liraglutide increases the thermogenic activation of BAT by CNS-GLP-1R signaling [24–26], little is known about the exact mechanisms underlying its activity in BAT activation and WAT browning (also termed brite or beige adipocytes). In this report, we focus on the function and mechanism of liraglutide in WAT browning.

WAT is characterized by a huge number of white adipocyte containing a large unilocular lipid droplet as well as few mitochondria, which is generally considered as insulation against cold and acts as the main site of metabolic energy storage in the form of TAG [38]. One feature of WAT browning is enhanced lipolysis [11], which is also known as the hydrolysis of TAG. ATGL and HSL are key enzymes involved in intracellular degradation of TAG [39]. We found ATGL and HSL were significantly up-regulated in iWAT and prWAT from liraglutide-treated mice (Figure 2A). The most important characteristics of brite fat are elevated expression of BAT marker genes. UCP1 is a specific marker of BAT because this protein is not remarkably expressed in WAT or in any other tissue, and it shortcuts the proton gradient in mitochondria and uncouples respiration from ATP synthesis to generate heat [11]. As a master regulator in white adipocyte differentiation, PPARγ is indispensable for brown adipocyte development, and PPARγ activators exerted a positive effect on BAT cells to result in increasing UCP1 mRNA and protein levels *in vivo* and *in vitro* [40]. The multilocular morphology of brown adipocyte is likely to be controlled by the regulation of lipid droplet growth. Cidea, which is only found in brown adipocytes, helps lipid droplets to grow by enabling one droplet to transfer its contents to another droplet [41]. As a transcriptional coregulator, PRDM16 has a pivotal role in the development of brite cells in s.c. WAT. Adipocyte-specific deletion of PRDM16 inhibited the induction of thermogenic activity in brite adipocytes following treated by cold exposure or β3 adrenergic agonist, and HFD-fed PRDM16-deficient mice tended to develop obesity [7, 42]. Excitedly, our results showed that liraglutide boosted mRNA and protein expression levels of BAT marker genes UCP1, PPARγ, Cidea and PRDM16 (Figure 2B and 2C). Mitochondria plays a key role in energy metabolism, and mitochondrial biogenesis enhancement is another characteristic feature of WAT browning [43]. As an interacting partner of PPARγ, transcriptional coactivator PGC1α is a master regulator of mitochondrial biogenesis and oxidative metabolism in BAT, which can also induce the expression of UCP1 and stimulate activation of BAT thermogenic [13]. TFAM is a major controller of mitochondrial mass, the deficiency of which in mice severely compromised activity of proteins in respiratory complexes I, III, and IV, resulting in BAT whitening [43]. CytoC is a heme protein that is essential for aerobic respiration and plays a key role in mitochondrial oxidative phosphorylation [44]. Our results revealed that PGC1α, TFAM and CytoC were up-regulated in iWAT and prWAT from mice treated by liraglutide (Figure 3A and 3B). Elevated mitochondrial numbers were also confirmed by immunostaining and IHC analysis (Figure 3C and 3D). It has been reported that liraglutide stimulates BAT thermogenesis and WAT browning by ICV injection after 24 h [24, 26]. However, in our study, we confirmed WAT browning could be induced with chronic liraglutide treatment for 12 weeks. *In vitro* studies were conducted to further address the biological effects of liraglutide on the formation of brown-like adipocytes in preadipocyte 3T3-L1. In line with *in vivo* data, we found a browning phenomenon of 3T3-L1 cells with liraglutide treatment, including greater numbers and smaller size lipid droplet, elevated expression of BAT and mitochondrial marker genes, and increased mitochondrial numbers (Figure 4). These results strongly support the notion that liraglutide promotes the process of WAT browning *in vivo* and *in vitro*.

There are two types of second messengers, cAMP and cGMP, which are important in transmitting hormonal signals from receptors on the cell membrane into the endochylema in adipocytes [19]. BAT research has focused mainly on cAMP that activates UCP1 expression via cAMP/PKA pathway or PKA independent cascade, in which, however, cGMP also plays an important role [19]. cGMP is synthesized by sGC upon activation by NO, and sGC stimulator promotes differentiation of human brown adipocytes as well as induces ‘browning’ of primary white adipocytes [35]. As the major downstream target of cGMP, PKGI plays a central role in BAT. BAT of PKGI-deficient mice has dramatically reduced expression levels of the thermogenic key protein UCP1, and global overexpression of PKGI results in upregulation of PGC1α and UCP1 in BAT [45]. As we known, those pathways involved in BAT always play important roles in WAT browning, for instance, the AMPK-mediated pathway [26, 46], so we thought NO/sGC/cGMP/PKGI signaling might involve in the process of WAT browning by liraglutide treatment. Our data show that liraglutide significantly increased expression of sGCβ and PKGI in WAT and adipocyte differentiation 3T3-L1 cells (Figure 5). It has been reported that NO/sGC/cGMP/PKGI pathway can also stimulate mitochondrial biogenesis [45], so the up-regulated sGCβ and PKGI by liraglutide was consistent with the results of its effect on enhanced mitochondrial biogenesis in Figures 3 and 4. These results indicate that the effects of liraglutide are mediated via NO/sGC/cGMP/PKGI signaling. In addition, to further identify the role of sGCβ in the liraglutide-induced browning pathway, we treated adipocytes with BAY 41-8543, a potent activator of sGCβ, or NS-2028, a selective inhibitor. We found activated expression of sGCβ significantly up-regulated cGMP downstream gene PKGI and BAT marker genes, but NS-2028 didn't obviously reduce the expression of sGCβ, therefore resulting in slightly down-regulating PKGI and BAT marker genes, we thought it was because of rare expression of sGCβ in white adipocytes (Figure 6). These findings strongly enforce our conclusion that liraglutide induces WAT browning via the sGC-mediated pathway. Although NO/sGC/cGMP/PKGI signaling is a key pathway in the process of WAT browning, the accurate molecular mechanisms by which NO/sGC/cGMP/PKGI signaling is activated during WAT browning remain to be explored.

In summary, we demonstrate the role of liraglutide in WAT browning *in vivo* and *in vitro*. Our findings provide evidence that liraglutide induces WAT browning by increasing expression of genes specific to BAT and mitochondrial biogenesis, which appeared to be mediated by sGC-dependent pathway. We also certify liraglutide contributes to its beneficial effects in metabolism on WAT browning with chronic s.c. treatment besides rapid ICV injection. This study expands our knowledge on the mechanism of liraglutide inducing WAT browning, and provides a theoretical support for clinical usage of liraglutide on obesity treatment.

## MATERIALS AND METHODS

### Ethics statement, mice

All animal experiments and procedures were approved by the Animal Ethical and Experimental Committee of the Tianjin Medical University Metabolic Diseases Hospital. 7-week-old male KKAy mice were purchased from HFK bioscience company (Beijing, China). All experimental mice were maintained on a 12:12 h light-dark cycle at 22°C under specific pathogen-free conditions in the animal facility at Tianjin Medical University (Tianjin, China). As a model of obesity, the mice were fed on a standard high fat rodent diet supplied by HFK bioscience company, food and water were provided ad libitum.

After a week of acclimation, mice were randomly divided into liraglutide group (*n* = 7, treated with final dose 400 μg/Kg body weight) and vehicle group (*n* = 7, treated with equivalent volume of PBS). Liraglutide and PBS were given s.c. injection respectively once daily at 16:00 pm for 12 weeks. The body weight of mice was recorded every day.

All mice treated with PBS or liraglutide were sacrificed by CO_2_ asphyxiation after 12 weeks. iWAT and prWAT were collected and divided into three specimens respectively. One was extracted RNA for qRT-PCR measurement, the second was isolated protein for western blot detection, and the third was fixed for Hematoxylin-Eosin (H&E) staining, immunostaining as well as IHC analyzed.

### Cell lines

Preadipocyte 3T3-L1 cells were cultured in DMEM (Hyclone, Thermo Fisher, Beijing, China) with 10% FBS, and were maintained in a humidified incubator containing 5% CO_2_ at 37°C.

### Reagents

Liraglutide was purchased from Novo Nordisk (Tianjin, China). BAY 41-8543 and NS-2028 was purchased from Cayman Chemical Company (Ann Arbor, MI, USA). Anti-β-actin for Western blot assay was purchased from Sigma Chemical (St.Louis, MO, USA). Anti-Cidea, Anti-PPARγ, Anti-UCP-1, Anti-CytoC, Anti-PGC1α, Anti-TFAM and Anti-PKGI for Western blot assay, Anti-sGCβ for Western blot and immunostaining assay, Anti-COX-IV for immunostaining and IHC assay were purchased from Protein Tech Group Ptglab (Wuhan, China).

### Quantitative RT-PCR

Total RNA was extracted from tissues with Trizol reagent (Invitrogen, Carlsbad, CA, USA). Total RNA was extracted from cells using an isolation kit (Omega Bio-Tek, Norcross, GA, USA). 1 μg total RNA of each sample was reverse-transcribed to cDNA with Reverse Transcription Kit (Thermo Fisher, Beijing, China). Quantitative PCR analyses for the mRNA expression were performed by using TransStart Green qPCR SuperMix (TransGen Biotech, Beijing, China) on the Light Cycler® 96 Real-Time PCR System (Roche Diagnostics Ltd, Mannheim, Germany), as the following parameters: 95°C for 30s followed by 40 cycles of 95°C for 5 s, 60°C for 5 s and 72°C for 20 s. The mRNA level of β-actin was used as an internal control. Relative expression was calculated using the comparative threshold cycle method.

The primer sequences used were listed in Supplementary Table S1.

### Western blot

Treated tissues and cells were lysed by RIPA Lysis Buffer (Beyotime, Shanghai, China), and the protein concentration was measured by BCA protein assay kit (Beyotime, Shanghai, China). The immunoblotting was performed as previously described [47]. Briefly, the protein was separated in 12% SDS denatured polyacrylamide gel and then transferred onto a polyvinylidene difluoride membrane. The membranes were blocked with 5% skim milk in Tris-buffered saline, pH 7.4, containing 0.1% Tween 20, at room temperature for 1 h, and were incubated with appropriate antibodies at 4°C overnight respectively. Membranes were washed and incubated with horseradish peroxidase (HRP)-conjugated secondary antibodies (1:3000, ZSGB-BIO, Beijing, China) according to the manufacturer's instructions. Finally, the protein of interest was visualized using Immobilon Western Chemiluminescent HRP Substrate (Millipore, Billerica, MA, USA).

### Histology

For adipocyte size analysis, one part of iWAT and prWAT was fixed with 4% (weight/vol) paraformaldehyde, and then the tissues were embedded in paraffin. Sections (4 μm thick) which had been deparaffinized and rehydrated were stained with H&E, and the size of adipocytes was determinated by computer image analysis as previously described [48]. For IHC analysis, after deparaffinization and rehydration, antigen retrieval was achieved by boiling in 10mM citrate buffer (pH 6.4) for 10 minutes in microwave. Endogenous peroxidase activity was quenched by incubating the slides in 3% (v/v) hydrogen peroxide in absolute methanol for 15 minutes, and was permeabilized in 1% TritonX-100 for 10 minutes. Blocking was performed in 10% goat serum in PBS for 15 minutes. A rabbit antibody to COX-IV was used as the primary antibody at a dilution of 1:100. After overnight incubation with the COX-IV antibody at 4°C, the slides were incubated with an anti-rabbit, HRP labeled polymer secondary antibody. Immunoreactivity was visualized with 3,3′-diaminobenzidine (ZSGB-BIO, Beijing, China). For blank control, all incubation steps were identical except that rabbit nonimmune IgG serum was used rather than the primary antibody (COX-IV). For tissue immunostaining experiment, all procedures were same as IHC analysis but no endogenous peroxidase activity blocking. After incubating with primary antibody of COX-IV or sGCβ overnight, the slides were serially incubated in Goat anti-rabbit Alexa Fluo488 (Invitrogen Corporation, Carlsbad, CA, USA). The images were acquired using the AV300-ASW confocal microscope (Olympus America Inc., Center Valley, USA). Image magnification: 100×.

### Cell differentiation and treatment

When preadipocyte 3T3-L1 cells reached approximately 90% confluence, for adipocyte differentiation, the cells were cultured in adipocyte-inducing medium (AIM, α-MEM containing 10% FBS, 0.5 μM dexamethasone, 0.25 mM methylisobutylxanthine, 5 μg/ml of insulin, and 50 μM indomethacin) for 3 days.

For liraglutide, BAY 41-8543 or NS-2028 treatment, cells were cultured in the presence of AIM for 3 days, and cells were then treated with different concentrations of liraglutide, 3 μM BAY 41-8543 or 100 nM NS-2028 in normal culturing medium (α-MEM containing 10% FBS).

### Oil red O staining

Treated adipocyte-inducing 3T3-L1 cells were gently washed twice with PBS, and then fixed in 4% paraformaldehyde for 10 min. Next, the cells were washed twice with deionized water, and then stained with 60% saturated oil red O for 5 min. For oil red O quantification, 4% IGEPAL CA 630 (Sigma) in isopropanol was added to each well, and then the plate was rocked on a shaker for 15 min. Light absorbance by the extracted dye was measured at 520 nm.

### Cell immunofluorescence

Adipocyte-inducing 3T3-L1 cells were treated with liraglutide for 3 days as described above. Cells were fixed in 4% paraformaldehyde and permeabilized by 1% TritonX-100. Following blocking with 1% bovine serum albumin, cells were serially incubated in rabbit anti-COX-IV and Goat anti-rabbit Alexa Fluo488 (Invitrogen Corporation, Carlsbad, CA, USA). The images were acquired using the AV300-ASW confocal microscope (Olympus America Inc., Center Valley, USA). Image magnification: 100×.

### Statistical analysis

The results are expressed as mean ± Standard Deviation (S.D.). A two-tailed student's *t* test was applied to analyze the differences between groups. ANOVA followed by Bonferroni multiple comparison pos*t*-tests was used for body weight comparisons between two independent groups. Statistical analysis was performed with SPSS software (version 17). Statistical differences were declared significant at *P* < 0.05 level. Statistically significant data are indicated by asterisks (*P* < 0.05 (*), *P* < 0.01(**), and *P* < 0.001(***).

## SUPPLEMENTARY MATERIALS



## References

[1] Ng M, Fleming T, Robinson M, Thomson B, Graetz N, Margono C, Mullany EC, Biryukov S, Abbafati C, Abera SF (2014). Global, regional, and national prevalence of overweight and obesity in children and adults during 1980–2013: a systematic analysis for the Global Burden of Disease Study 2013. Lancet.

[2] Kissebah AH, Freedman DS, Peiris AN (1989). Health risks of obesity. Med Clin North Am.

[3] Kopelman P (2007). Health risks associated with overweight and obesity. Obes Rev.

[4] Ahima RS, Lazar MA (2013). The health risk of obesity—better metrics imperative. Science.

[5] Gesta S, Tseng YH, Kahn CR (2007). Developmental origin of fat: tracking obesity to its source. Cell.

[6] Speakman JR (2010). FTO effect on energy demand versus food intake. Nature.

[7] Jeremic N, Chatuverdi P, Tyagi SC (2016). Browning of White Fat: Novel Insight into Factors, Mechanisms and Therapeutics. J Cell Physiol.

[8] Zhang G, Sun Q, Liu C (2016). Influencing Factors of Thermogenic Adipose Tissue Activity. Front Physiol.

[9] Enerback S, Jacobsson A, Simpson E, Guerra C, Yamashita H, Harper M, Kozak L (1997). Mice lacking mitochondrial uncoupling protein are cold-sensitive but not obese. Nature.

[10] Feldmann HM, Golozoubova V, Cannon B, Nedergaard J (2009). UCP1 ablation induces obesity and abolishes diet-induced thermogenesis in mice exempt from thermal stress by living at thermoneutrality. Cell Metab.

[11] Poekes L, Lanthier N, Leclercq IA (2015). Brown adipose tissue: a potential target in the fight against obesity and the metabolic syndrome. Clin Sci.

[12] Townsend K, Tseng Y (2015). Of mice and men: novel insights regarding constitutive and recruitable brown adipocytes. Int J Obes Suppl.

[13] Kim SH, Plutzky J (2016). Brown fat and browning for the treatment of obesity and related metabolic disorders. Diabetes Metab J.

[14] Tharp KM, Stahl A (2015). Bioengineering beige adipose tissue therapeutics. Front Endocrinol.

[15] Lo KA, Sun L (2013). Turning WAT into BAT: a review on regulators controlling the browning of white adipocytes. Biosci Rep.

[16] Seale P, Conroe HM, Estall J, Kajimura S, Frontini A, Ishibashi J, Cohen P, Cinti S, Spiegelman BM (2011). Prdm16 determines the thermogenic program of subcutaneous white adipose tissue in mice. J Clin Invest.

[17] Ohno H, Shinoda K, Spiegelman BM, Kajimura S (2012). PPARγ agonists induce a white-to-brown fat conversion through stabilization of PRDM16 protein. Cell Metab.

[18] Boström P, Wu J, Jedrychowski MP, Korde A, Ye L, Lo JC, Rasbach KA, Boström EA, Choi JH, Long JZ (2012). A PGC1-α-dependent myokine that drives brown-fat-like development of white fat and thermogenesis. Nature.

[19] Pfeifer A, Hoffmann LS (2015). Brown, beige, and white: the new color code of fat and its pharmacological implications. Annu Rev Pharmacol Toxicol.

[20] Drucker DJ (2007). The role of gut hormones in glucose homeostasis. J Clin Invest.

[21] Holst JJ (2007). The physiology of glucagon-like peptide 1. Physiol Rev.

[22] Torekov S, Madsbad S, Holst JJ (2011). Obesity–an indication for GLP-1 treatment? Obesity pathophysiology and GLP-1 treatment potential. Obes Rev.

[23] Russell-Jones D (2009). Molecular, pharmacological and clinical aspects of liraglutide, a once-daily human GLP-1 analogue. Mol Cell Endocrinol.

[24] Lockie SH, Heppner KM, Chaudhary N, Chabenne JR, Morgan DA, Veyrat-Durebex C, Ananthakrishnan G, Rohner-Jeanrenaud F, Drucker DJ, DiMarchi R, Rahmouni K, Oldfield BJ, Tschop MH (2012). Direct control of brown adipose tissue thermogenesis by central nervous system glucagon-like peptide-1 receptor signaling. Diabetes.

[25] Kooijman S, Wang Y, Parlevliet ET, Boon MR, Edelschaap D, Snaterse G, Pijl H, Romijn JA, Rensen PC (2015). Central GLP-1 receptor signalling accelerates plasma clearance of triacylglycerol and glucose by activating brown adipose tissue in mice. Diabetologia.

[26] Beiroa D, Imbernon M, Gallego R, Senra A, Herranz D, Villarroya F, Serrano M, Fernø J, Salvador J, Escalada J (2014). GLP-1 agonism stimulates brown adipose tissue thermogenesis and browning through hypothalamic AMPK. Diabetes.

[27] Astrup A, Rössner S, Van Gaal L, Rissanen A, Niskanen L, Al Hakim M, Madsen J, Rasmussen MF, Lean ME, Group NS (2009). Effects of liraglutide in the treatment of obesity: a randomised, double-blind, placebo-controlled study. Lancet.

[28] Marre M, Shaw J, Brändle M, Bebakar WW, Kamaruddin NA, Strand J, Zdravkovic M, Le Thi T, Colagiuri S (2009). Liraglutide, a once-daily human GLP-1 analogue, added to a sulp+++honylurea over 26 weeks produces greater improvements in glycaemic and weight control compared with adding rosiglitazone or placebo in subjects with Type 2 diabetes (LEAD-1 SU). Diabet Med.

[29] Secher A, Jelsing J, Baquero AF, Hecksher-Sørensen J, Cowley MA, Dalbøge LS, Hansen G, Grove KL, Pyke C, Raun K (2014). The arcuate nucleus mediates GLP-1 receptor agonist liraglutide-dependent weight loss. J Clin Invest.

[30] Fransson L, dos Santos C, Wolbert P, Sjöholm Å, Rafacho A, Ortsäter H (2014). Liraglutide counteracts obesity and glucose intolerance in a mouse model of glucocorticoid-induced metabolic syndrome. Diabetol Metab Syndr.

[31] Heppner KM, Marks S, Holland J, Ottaway N, Smiley D, Dimarchi R, Perez-Tilve D (2015). Contribution of brown adipose tissue activity to the control of energy balance by GLP-1 receptor signalling in mice. Diabetologia.

[32] Morak M, Schmidinger H, Riesenhuber G, Rechberger GN, Kollroser M, Haemmerle G, Zechner R, Kronenberg F, Hermetter A (2012). Adipose triglyceride lipase (ATGL) and hormone-sensitive lipase (HSL) deficiencies affect expression of lipolytic activities in mouse adipose tissues. Mol Cell Proteomics.

[33] Haas B, Mayer P, Jennissen K, Scholz D, Berriel Diaz M, Bloch W, Herzig S, Fassler R, Pfeifer A (2009). Protein kinase G controls brown fat cell differentiation and mitochondrial biogenesis. Sci Signal.

[34] Jennissen K, Siegel F, Liebig-Gonglach M, Hermann MR, Kipschull S, van Dooren S, Kunz WS, Fassler R, Pfeifer A (2012). A VASP-Rac-soluble guanylyl cyclase pathway controls cGMP production in adipocytes. Sci Signal.

[35] Hoffmann LS, Etzrodt J, Willkomm L, Sanyal A, Scheja L, Fischer AW, Stasch JP, Bloch W, Friebe A, Heeren J, Pfeifer A (2015). Stimulation of soluble guanylyl cyclase protects against obesity by recruiting brown adipose tissue. Nat Commun.

[36] Ryan D, Acosta A (2015). GLP-1 receptor agonists: Nonglycemic clinical effects in weight loss and beyond. Obesity.

[37] Kanoski SE, Fortin SM, Arnold M, Grill HJ, Hayes MR (2011). Peripheral and central GLP-1 receptor populations mediate the anorectic effects of peripherally administered GLP-1 receptor agonists, liraglutide and exendin-4. Endocrinology.

[38] Warner A, Mittag J (2016). Breaking BAT: can browning create a better white?. J Endocrinol.

[39] Chaves VE, Frasson D, Kawashita NH (2011). Several agents and pathways regulate lipolysis in adipocytes. Biochimie.

[40] Forest C, Joffin N, Jaubert AM, Noirez P (2016). What induces watts in WAT?. Adipocyte.

[41] Barneda D, Planas-Iglesias J, Gaspar ML, Mohammadyani D, Prasannan S, Dormann D, Han GS, Jesch SA, Carman GM, Kagan V (2015). The brown adipocyte protein CIDEA promotes lipid droplet fusion via a phosphatidic acid-binding amphipathic helix. Elife.

[42] Hilton C, Karpe F, Pinnick KE (2015). Role of developmental transcription factors in white, brown and beige adipose tissues. Biochim Biophys Acta.

[43] Cedikova M, Kripnerová M, Dvorakova J, Pitule P, Grundmanova M, Babuska V, Mullerova D, Kuncova J (2016). Mitochondria in White, Brown, and Beige Adipocytes. Stem Cells Int.

[44] Babbitt SE, Sutherland MC, San Francisco B, Mendez DL, Kranz RG (2015). Mitochondrial cytochrome c biogenesis: no longer an enigma. Trends Biochem Sci.

[45] Hoffmann LS, Larson CJ, Pfeifer A (2016). cGMP and Brown Adipose Tissue. Handb Exp Pharmacol.

[46] Lone J, Choi JH, Kim SW, Yun JW (2016). Curcumin induces brown fat-like phenotype in 3T3-L1 and primary white adipocytes. J Nutr Biochem.

[47] Zhu E, Wang X, Zheng B, Wang Q, Hao J, Chen S, Zhao Q, Zhao L, Wu Z, Yin Z (2014). miR-20b suppresses Th17 differentiation and the pathogenesis of experimental autoimmune encephalomyelitis by targeting RORγt and STAT3. J Immunol.

[48] Chen HC, Farese RV (2002). Determination of adipocyte size by computer image analysis. J Lipid Res.

