# Supplementary material for: Liraglutide suppresses obesity and induces brown fat-like phenotype via Soluble Guanylyl Cyclase mediated pathway in vivo and in vitro
Source: Oncotarget. 2016 Nov 7;7(49):81077–89. doi: 10.18632/oncotarget.13189 (PMC5348377; doi:10.18632/oncotarget.13189)
Supplement: Supplementary file 1 [file oncotarget-07-81077-s001.pdf]

Liraglutide suppresses obesity and induces brown fat-like phenotype via Soluble Guanylyl Cyclase mediated pathway *in vivo* and *in vitro*

Supplementary Materials

Supplementary Table S1: Primer for qRT-PCR analyses to mRNA sequences in this article

Name	Sequence
**Primer for qRT-PCR analyses to mRNA**	
ATGL-F	5′-TAGCTAACAGTTGGGCTTCAC-3′
ATGL-R	5′- CAGAGAGAACAGAGCAGCTTAC-3′
HSL-F	5′-ACGGATACCGTAGTTTGGTGC-3′
HSL-R	5′-TCCAGAAGTGCACATCCAGGT-3′
Cidea-F	5′-CCTAGCACCAAAGGCTGGTT-3′
Cidea-R	5′-GTATCCACGCAGTTCCCACA-3′
PPARγ-F	5′-CTTGACAGGAAAGACAACGG-3′
PPARγ-R	5′-GCTTCTACGGATCGAAACTG-3′
PRDM16-F	5′-TCCCACCAGACTTCGAGCTA-3′
PRDM16-R	5′-CAAAGTCGGCCTCCTTCAGT-3′
UCP-1-F	5′-CTGCCAGGACAGTACCCAAG-3′
UCP-1-R	5′-TCAGCTGTTCAAAGCACACAAA-3′
CytoC-F	5′-GCTACCCATGGTCTCATCGTG-3′
CytoC-R	5′-CATCATCATTAGGGCCATCCT-3′
PGC1α-F	5′-TGTGTGCTGTGTGTCAGAGT-3′
PGC1α-R	5′-TGGTCGCTACACCACTTCAA-3′
TFAM-F	5′-GTCCATAGGCACCGTATTGCG-3′
TFAM-R	5′-CCCATGCTGGAAAAACACTTCG-3′
sGCβ1-F	5′-ACAGGTGTCTCATGTCTCCA-3′
sGCβ1-R	5′-GTGCTCCTTGCTTGACACAC-3′
PKGI-F	5′-CTGCCTTCTTCGCCAACCT-3′
PKGI-R	5′-CCCCTGCATGATCTGCTTCTC-3′
β-actin-F	5′-AAGACCTCTATGCCAACACAG-3′
β-actin-R	5′-GGAGGAGCAATGATCTTGATC-3′
